# The association between platelet-to-lymphocyte ratio with mortality among patients suffering from acute decompensated heart failure

**DOI:** 10.1186/s12872-021-02260-7

**Published:** 2021-09-18

**Authors:** Maryam Heidarpour, Sepideh Bashiri, Mehrbod Vakhshoori, Kiyan Heshmat-Ghahdarijani, Farbod Khanizadeh, Shaghayegh Ferdowsian, Davood Shafie

**Affiliations:** 1 Isfahan Endocrine and Metabolism Research Center, Isfahan University of Medical Sciences, Isfahan, Iran; 2 Cardiac Rehabilitation Research Center, Isfahan Cardiovascular Research Institute, Isfahan University of Medical Sciences, Isfahan, Iran; 3 Heart Failure Research Center, Cardiovascular Research Institute, Isfahan University of Medical Sciences, Isfahan, Iran; 4 Heart Failure Research Center, Cardiovascular Research Institute, Isfahan University of Medical Sciences, Isfahan, Iran; 5 Insurance Research Center, Tehran, Iran

**Keywords:** Heart failure, Lymphocytes, Blood platelets, Mortality

## Abstract

**Background:**

Platelet-to-lymphocyte ratio (PLR) is an inflammation index suggested to have the prognostic capability in heart failure (HF). We sought to investigate the association of PLR with cardiovascular disease (CVD) mortality and creatinine (Cr) rise among Iranian individuals suffering from acute decompensated HF (ADHF).

**Methods:**

This retrospective cohort study was in the context of the Persian Registry Of cardioVascular diseasE/Heart Failure (PROVE/HF) study. 405 individuals with ADHF admitted to the emergency department were recruited from April 2019 to March 2020. PLR was calculated by division of platelet to absolute lymphocyte counts and categorized based on quartiles. We utilized the Kaplan–Meier curve to show the difference in mortality based on PLR quartiles. Cr rise was defined as the increment of at least 0.3 mg/dl from baseline. Cox proportional hazard ratio (HR) was used to investigate the association of PLR with CVDs mortality.

**Results:**

Mean age of participants was 65.9 ± 13.49 years (males: 67.7%). The mean follow-up duration was 4.26 ± 2.2 months. CVDs mortality or re-hospitalization was not significantly associated with PLR status. Multivariate analysis of PLR quartiles showed a minimally reduced likelihood of CVDs death in 2nd quartile versus the first one (HR 0.40, 95% confidence interval (CI) 0.16–1.01, P = 0.054). Cr rise had no remarkable relation with PLR status in neither model.

**Conclusion:**

PLR could not be used as an independent prognostic factor among ADHF patients. Several studies are required clarifying the exact utility of this index.

## Introduction

Heart failure (HF) is a major global public health problem with increased prevalence with aging [1]. It has been reported that more than 5 million people in the USA suffer from HF, and this number rises to be 23 million individuals all over the world [2]. This disease significantly affects the quality of life among the elderly population. Regardless of the progress in heart failure management in recent years, the long-term clinical outcomes are still poor [3, 4].

An appropriate approach toward HF management requires knowledge of mechanisms and precipitating factors contributing to its development [5]. Recently, biomarkers have been introduced to help physicians predicting clinical outcomes and improve the current knowledge about the precise pathophysiology of cardiovascular disorders, including HF [6–8]. Some of these biomarkers' prognostic values have been extensively investigated over the past decades [7, 9]. Inflammation has untoward effects on cardiac function and contributes to HF development [10, 11]. Evidences gathered over the past 30 years from both in vitro and in vivo studies gave recognized multiple immune system mechanisms contributing to the onset of systolic dysfunction and progressive structural changes in patients with chronic HF [12, 13].

On the other hand, inflammation plays an essential role in the onset and progression of acute kidney injury [14]. Moreover, acute renal impairment is of great importance in patients hospitalized due to HF. Cardiac and renal functions are interrelated through various ways that derangement in one’s function can affect the other [15].

Platelet to lymphocyte ratio (PLR) is a novel inflammatory biomarker. Several studies demonstrated the utility of PLR as a predictor for the outcome of a wide range of diseases in which inflammation plays an important role, including malignancies, cardiovascular events, and renal diseases [16–18]. Furthermore, some studies have investigated the PLR in predicting mortality in acute HF patients with controversial results [19–21]. On the other hand, Zheng and colleagues demonstrated that higher and lower PRLs were associated with increased all-cause mortality in acute kidney injury [18].

Considering the importance of the prognosis of patients with acute HF, we sought to investigate the relationship between PLR with cardiovascular disease (CVD) mortality and readmission rates as well as serum creatinine levels among Iranian hospitalized patients suffering from acute decompensated HF (ADHF).

## Materials and methods

This retrospective single-center cohort study was done in the context of the Persian Registry Of cardioVascular diseasE/Heart Failure (PROVE/HF) study [22]. The exact methodological explanation is described elsewhere. Briefly, this database was launched in March 2015 and continuously gathering data of Iranian HF patients. A questionnaire containing 27 items, including demographic characteristics, underlying chronic diseases, laboratory, para-clinic information, and recruited patients' data, were collected. From April 2019 to March 2020, any individual aged at least 18 years suffered from ADHF admitted in one of the tertiary governmental heart center (Charman hospital, Isfahan, Iran) was eligible for enrollment in our study. We included those patients admitted to the emergency department. We did not differentiate subjects according to the type of ADHF. All patients with previously proved HF or de novo HF referred with signs and symptoms of decompensation approved by skillful cardiologists had an equal chance to be recruited. The presence of previously untreated chronic comorbidities, including liver diseases, malignancy, severe infection, current usage of corticosteroid or chemotherapeutic agents, and unwillingness to participate in the project or incompleteness of their medical profiles, were considered as exclusion criteria. We further excluded admitted HF individuals with stable conditions. Each participant was fully explained the aims of the project by the principal investigator. Any probable questions were answered thoroughly, and all patients were told that no legal issues were attributed to them in terms of exclusion from the study. The ethics committee affiliated with Isfahan University of Medical Sciences (IUMS) proved this study (IR.MUI.MED.REC.1399.538).

Patients who fulfilled the inclusion criteria were recruited consecutively during our pre-defined study period. Finally, a total of 405 individuals were included in the study. Data about age, gender (male/female), smoking habits, ischemic heart disease, diabetes mellitus, hypertension, stroke, kidney diseases, and chronic obstructive pulmonary (COPD) disease were assessed through a questionnaire. Body mass index (BMI) was calculated by division of weight (in kilograms) over height (in squared meters) (kg/m^2^). All subjects were also asked about consumption of beta-blockers, angiotensin-converting enzyme inhibitors (ACEIs), angiotensin receptor blockers (ARBs), diuretics, statins, and oral anticoagulants before current hospitalization. At admission time, heart rates, blood pressure (BP) indices, including systolic BP (SBP), diastolic BP (DBP) as well as left ventricular ejection fraction (LVEF), were assessed. Additionally, a blood sample was taken from each participant and blood indices including hemoglobin (g/dl), white blood cell count (10^6^/l), neutrophils (%), lymphocytes(%), platelets (10^9^/l), sodium (mEq/l), potassium (mEq/l), blood urea nitrogen (BUN) (mg/dl) and creatinine (Cr) (mg/dl) were measured. We measured Cr levels at least two times, and subtraction on the discharge date from the admission date was calculated to assess the presence/absence of increased Cr levels. Subsequently, Cr rise was defined as an increase in serum Cr of at least 0.3 mg/dl from the baseline. The aforementioned medication consumption status was also assessed at discharge time. The participants were followed within the next six months after discharge via telephone surveys every three months. Proper documents were requested from patients/first-degree relatives in terms of CVDs death or re-hospitalization. Patients who died in the context of other etiologies rather than cardiovascular events were discarded from the study.

### Statistical analysis

Continuous and categorical variables were reported as mean ± standard deviation (SD) and frequency (percentage), respectively. To examine the relationship between nominal and numerical variables, chi-square and t-test/analysis of variance (ANOVA) were utilized, respectively. We used Kaplan–Meier curves to show the difference between CVDs mortality according to PLR quartiles and the presence/absence of Cr rise. In order to examine the potential differences between each pre-defined continuous and categorical group, post hoc Bonferroni test was implemented. Finally, univariate and multivariate-adjusted models based on variables with significant differences according to PLR quartiles were implemented to investigate the hazard ratio (HR) and odds ratio (OR) of CVD mortality and Cr rise based on PLR quartiles, respectively. Also, we assessed the HR of CVDs mortality based on Cr rise. All analyses were performed using Statistical Package for Social Sciences (SPSS Inc., version 22.0, Chicago, IL, USA). We considered the P-values of less than 0.05 as statistically significant.

## Results

The mean age of all study population was 65.9 ± 13.49 years. Males were the dominant group (67.7%). The mean follow-up time was 4.26 ± 2.2 months. General characteristics of participants according to PLR quartiles are provided in Table 1. COPD was differed significantly based on the PLR quartiles (P = 0.002). Our post hoc analysis showed that patients in the lowest quartile had a higher prevalence of COPD than in the highest quartile. In terms of laboratory data, those patients with higher PLR quartiles revealed difference in terms of hemoglobin (P = 0.021), WBC (P < 0.001), neutrophils (P < 0.001), lymphocytes (P < 0.001), platelets (P < 0.001) and sodium (P = 0.001). Post hoc analysis showed individuals within the highest PLR quartile had significantly lower means of WBC (3.4 ± 0.6*10^6^/l vs. 8.1 ± 3.7 * 10^6^/l, P < 0.001), lymphocytes (15.1 ± 6.3% vs. 28.1 ± 10.3%, P < 0.001) and sodium (134.76 ± 4.4 mEq/l vs. 137.42 ± 7 mEq/l, P < 0.001) and higher levels of neutrophils (76 ± 8.1% vs. 60.8 ± 14%, P < 0.001) and platelets (227.97 ± 78.8 * 10^9^/l vs. 160.23 ± 65.6 * 10^9^/l, P < 0.001) in comparison to the first quartile. Hemoglobin status was not different in the 4th quartile versus the 1st quartile (13.23 ± 3 g/dl vs. 14.27 ± 3 g/dl, P = 0.093). Pre-admission or discharge drug history were uniformly distributed among the study population regardless of PLR quartiles.

**Table 1:** General and laboratory characteristics and drug history of the study population according to PLR quartiles

Variables	Total	PLR quartiles				P
	(n = 405)	Q1 (PLR ≤ 118) (n = 100)	Q2 (119 < PLR < 198) (n = 103)	Q3 (198 ≤ PLR < 268) (n = 101)	Q4 (PLR ≥ 268) (n = 101)	
Age(years)	65.9 ± 13.49	65.3 ± 13.15	66.5 ± 12.8	65.7 ± 12.91	66.25 ± 15.13	0.913
Males (%)	274 (67.7)	72 (72)	68 (66)	64 (63.4)	70 (69.3)	0.580
BMI(kg/m^2^)	26.85 ± 6.2	26.45 ± 6.9	27.49 ± 7.2	27.32 ± 6	26.32 ± 3.9	0.417
Heart rate (beats per minute)	85.28 ± 19.5	83.64 ± 20.3	86.81 ± 19.2	85.22 ± 18.7	85.41 ± 19.8	0.722
Ischemic heart disease (%)	358 (88.4)	89 (89)	90 (87.4)	89 (88.1)	90 (89.1)	0.978
Diabetes mellitus (%)	170 (42)	42 (42)	38 (36.9)	50 (49.5)	40 (39.6)	0.299
Hypertension (%)	194 (47.9)	47 (47)	51 (49.5)	50 (49.5)	46 (45.5)	0.926
Stroke (%)	18 (4.4)	4 (4)	5 (4.9)	4 (4)	5 (5)	0.977
Kidney diseases (%)	179 (44.2)	44 (44)	47 (45.6)	46 (45.5)	42 (41.6)	0.932
COPD (%)	142 (35.1)	41 (41)^d^	35 (34)	45 (44.6)	21 (20.8)	0.002
Smoking status (%)	152 (37.5)	38 (38)	36 (35)	37 (36.6)	41 (40.6)	0.864
Systolic blood pressure (mmHg)	121.01 ± 26.9	122.79 ± 26.9	121.01 ± 23.8	119.58 ± 24.2	120.68 ± 25.5	0.839
Diastolic blood pressure (mmHg)	75.67 ± 15.68	76.26 ± 16.6	75.54 ± 15.2	75.76 ± 14.1	75.14 ± 16.8	0.967
Left ventricular ejection fraction (%)						
< 30	299 (73.8)	73 (73)	70 (68)	78 (77.2)	78 (77.2)	0.391
30–40	61 (15.1)	18 (18)	17 (16.5)	12 (11.9)	14 (13.9)	
40–50	27 (6.7)	3 (3)	9 (8.7)	9 (8.9)	6 (5.9)	
> 50	18 (4.4)	6 (6)	7 (6.8)	2 (2)	3 (3)	
Hemoglobin (g/dl)	13.59 ± 3	14.27 ± 3^b^	13.79 ± 2.3	13.08 ± 3.5	13.23 ± 3	0.021
WBC (10^6^/l)	5.4 ± 3.1	8.1 ± 3.7^a,b,c^	5.8 ± 2.9	4.1 ± 2.1	3.4 ± 0.6	< 0.001
Neutrophils (%)	68 ± 12.3	60.8 ± 14^a,b,c^	68.4 ± 10.6	66.8 ± 11.1	76 ± 8.1	< 0.001
Lymphocytes (%)	22 ± 9.5	28.1 ± 10.3^a,b,c^	23 ± 9.2	22 ± 6.9	15.1 ± 6.3	< 0.001
Platelets (10^9^/l)	190.45 ± 68.9	160.23 ± 65.6^b,c^	175.28 ± 49.4	198.32 ± 59.9	227.97 ± 78.8	< 0.001
Sodium (mEq/l)	136.07 ± 4.8	137.42 ± 7^b,c^	136.5 ± 2.9	135.6 ± 3.8	134.76 ± 4.4	0.001
Potassium (mEq/l)	3.97 ± 0.5	3.98 ± 0.5	3.93 ± 0.5	3.97 ± 0.5	4.01 ± 0.5	0.762
Blood urea nitrogen (mg/dl)	83.36 ± 52.2	82.61 ± 60.9	84.22 ± 45.8	80.41 ± 48.8	86.2 ± 52.9	0.881
Creatinine (mg/dl)	1.56 ± 1.2	1.42 ± 0.7	1.50 ± 0.5	1.47 ± 0.7	1.84 ± 2.2	0.077
Drug history						
Pre-admission						
Beta blocker (%)	158 (39)	44 (44)	40 (38.8)	34 (33.7)	40 (39.6)	0.517
ACEIs/ARBs (%)	200 (49.4)	47 (47)	46 (44.7)	54 (53.5)	53 (52.5)	0.531
Diuretics (%)	271 (66.9)	62 (62)	72 (69.9)	72 (71.3)	65 (64.4)	0.444
Statins (%)	205 (50.6)	53 (53)	48 (46.6)	55 (54.5)	49 (48.5)	0.645
Oral anti-coagulants (%)	108 (26.7)	23 (23)	31 (30.1)	30 (29.7)	24 (23.8)	0.528
Discharge						
Beta blocker (%)	273 (67.4)	67 (67)	72 (69.9)	68 (67.3)	66 (65.3)	0.920
ACEIs/ARBs (%)	192 (47.4)	49 (49)	50 (48.5)	49 (48.5)	44 (43.6)	0.849
Diuretics (%)	326 (80.5)	82 (82)	87 (84.5)	78 (77.2)	79 (78.2)	0.532
Oral anti-coagulants (%)	113 (27.9)	20 (20)	29 (28.2)	37 (36.6)	27 (26.7)	0.072

*PLR* platelet to lymphocyte ratio, *BMI* body mass index, *COPD* chronic obstructive pulmonary disease, *ACEI* angiotensin-converting enzyme inhibitors, *ARB* angiotensin receptor blockers, *WBC* white blood cells

^a^P < 0.05 compared with quartile 2 resulted from post hoc Bonferroni test

^b^P < 0.05 compared with quartile 3 resulted from post hoc Bonferroni test

^c^P < 0.05 compared with quartile 4 resulted from post hoc Bonferroni test

^d^P < 0.05 compared with quartile 4 resulted from post hoc test with Bonferroni correction

44 (10.9%) deaths in the context of cardiovascular events occurred during the follow-up duration. CVDs mortality and re-hospitalization during pre-defined follow-up duration are demonstrated in Table 2. Our findings were insignificant in terms of CVDs death or readmission according to different categories of PLR. Also, we found no significant relationship in terms of CVDs mortality or re-hospitalization according to Cr rise status.

**Table 2:** Distribution of cardiovascular disease mortality and re-hospitalization of study population according to PLR quartiles and creatinine rise

Variables	Total	PLR quartiles				P	Creatinine rise		P
	(n = 405)	Q1 (PLR ≤ 118) (n = 100)	Q2 (119 < PLR < 198) (n = 103)	Q3 (198 ≤ PLR < 268) (n = 101)	Q4 (PLR ≥ 268) (n = 101)		Negative (n = 290)	Positive (n = 115)	
CVD mortality (%)	44 (10.9)	14 (14)	8 (7.8)	11 (10.9)	11 (10.9)	0.565	36 (12.4)	8 (7)	0.112
Re-hospitalization (%)	98 (24.2)	24 (24)	28 (27.2)	30 (29.7)	16 (15.8)	0.111	68 (23.4)	30 (26.1)	0.576

*PLR* platelet to lymphocyte ratio, *CVD* cardiovascular disease

Table 3 presents the general characteristics of the study population based on mortality/survival status. PLR was distributed uniformly between deceased and alive patients (127.80 ± 77.5 vs. 129.03 ± 108.6, P = 0.942). In comparison to alive participants, deceased ones had significantly higher prevalence of COPD (70.5% vs. 30.7%, P < 0.001), higher neutrophil percentages (73 ± 9.5% vs. 67.4 ± 12.5%, P = 0.005) and BUN levels (103.73 ± 61.09 mg/dl vs. 80.88 ± 50.63 mg/dl, P = 0.006). On the other hand, the mean of platelets, lymphocytes, and sodium were remarkably higher among survived patients rather than dead ones.

**Table 3:** General and laboratory characteristics of the study population according to mortality or survival status

Variables	Mortality (n = 44)	Survival (n = 361)	P
Age(years)	67.57 ± 15.58	65.79 ± 13.22	0.410
Males (%)	28 (63.6)	246 (68.1)	0.546
BMI(kg/m^2^)	25.68 ± 4.8	27.05 ± 6.2	0.165
Heart rate (beats per minute)	83.04 ± 16	85.56 ± 19.9	0.420
Ischemic heart disease (%)	37 (84.1)	321(88.9)	0.345
Diabetes mellitus (%)	21 (47.7)	149 (41.3)	0.413
Hypertension (%)	16 (36.4)	178 (49.3)	0.105
Stroke (%)	1 (2.3)	17 (4.7)	0.459
Kidney diseases (%)	19 (43.2)	160 (44.3)	0.886
COPD (%)	31 (70.5)	111 (30.7)	< 0.001
Smoking status (%)	14 (31.8)	138 (38.2)	0.407
Systolic blood pressure (mmHg)	116.7 ± 19.5	121.54 ± 25.6	0.228
Diastolic blood pressure (mmHg)	72.29 ± 12.5	76.08 ± 15.9	0.130
Left ventricular ejection fraction (%)			
< 30	34 (77.3)	265 (73.4)	0.338
30–40	3 (6.8)	58 (16.1)	
40–50	4 (9.1)	23 (6.4)	
> 50	3 (6.8)	15 (4.2)	
Hemoglobin (g/dl)	13.12 ± 2.3	13.65 ± 3.1	0.279
WBC (10^6^/l)	5.9 ± 3.2	5.3 ± 3.1	0.199
Neutrophils (%)	73 ± 9.5	67.4 ± 12.5	0.005
Lymphocytes (%)	17.9 ± 7.3	22.5 ± 9.6	0.002
Platelets (10^9^/l)	170.38 ± 61.8	192.89 ± 69.4	0.041
Platelet-to-lymphocyte ratio	127.80 ± 77.5	129.03 ± 108.6	0.942
Sodium (mEq/l)	134.7 ± 5.4	136.23 ± 4.7	0.049
Potassium (mEq/l)	3.96 ± 0.4	3.98 ± 0.5	0.862
Blood urea nitrogen (mg/dl)	103.73 ± 61.09	80.88 ± 50.63	0.006
Creatinine (mg/dl)	1.66 ± 0.7	2.4 ± 1	0.643

**Fig. 1:**
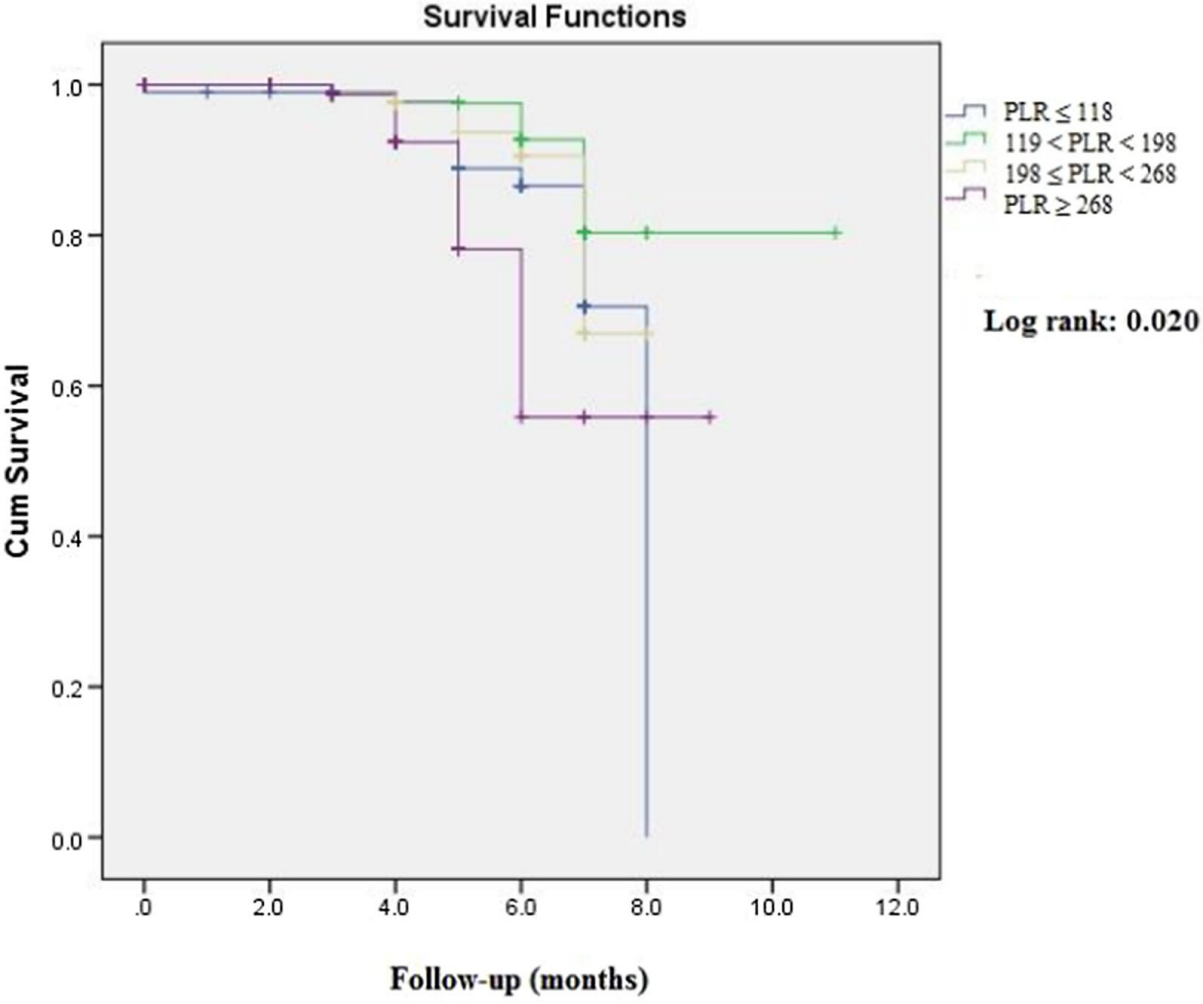
Kaplan–Meier survival curve of study population according to PLR quartiles

Kaplan–Meier curve, as shown in Fig. 1, revealed a significant difference in terms of CVDs mortality (Log-rank: 0.020). Pairwise comparison showed that patients within the highest PLR quartile deceased more frequently than those in the second quartile (chi-square: 8.68, P = 0.003). There was no significant difference between the other quartiles.

Table 4 shows the univariate and multivariable-adjusted hazard ratio and the odds ratio for CVDs mortality, and the Cr rise based on the PLR quartiles, respectively. Although patients with higher PLR levels had a higher likelihood of death than the first quartile in the univariate model, this relation was insignificant (HR 2.10, 95% CI 0.92—4.77, P = 0.075). Interestingly, multivariate-adjusted HR showed that patients in the second PLR quartile (119 < PLR < 198) had a marginally reduced likelihood of CVDs death in comparison to the reference group (PLR ≤ 118) (HR 0.40, 95% CI 0.16—1.01, P = 0.054). In terms of Cr rise, no remarkable association had been found according to PLR quartiles stratification, neither in univariate nor in multivariate models.

**Table 4:** Univariate and multivariable-adjusted Cox regression hazard ratio and odds ratio of cardiovascular disease mortality and creatinine rise according to PLR quartiles

Variables	Models	PLR quartiles				P†	Pƪ	P¶
		Quartile 1 (PLR ≤ 118)	Quartile 2 (119 < PLR < 198)	Quartile 3 (198 ≤ PLR < 268)	Quartile 4 (PLR ≥ 268)			
CVD mortality	Univariate	1.00	0.54 (0.22–1.29)	0.87 (0.39–1.93)	2.10 (0.92–4.77)	0.169	0.741	0.075
	Multivariate*	1.00	0.40 (0.16–1.01)	0.61 (0.23–1.56)	1.12 (0.34–3.72)	0.054	0.305	0.845
Creatinine rise	Univariate	1.00	0.69 (0.36–1.32)	1.36 (0.74–2.48)	1.08 (0.59–2)	0.273	0.310	0.790
	Multivariate*	1.00	0.79 (0.39–1.63)	1.60 (0.76–3.34)	1.44 (0.60–3.44)	0.538	0.211	0.413

*CVD* cardiovascular diseases, *PLR* platelet to lymphocyte ratio

P†: P-value resulted from the comparison between quartile 1 and quartile 2

Pƪ: P-value resulted from the comparison between quartile 1 and quartile 3

P¶: P-value resulted from the comparison between quartile 1 and quartile 4

*Adjusted for chronic obstructive pulmonary disease, hemoglobin, white blood cells, neutrophils, and sodium

**Fig. 2:**
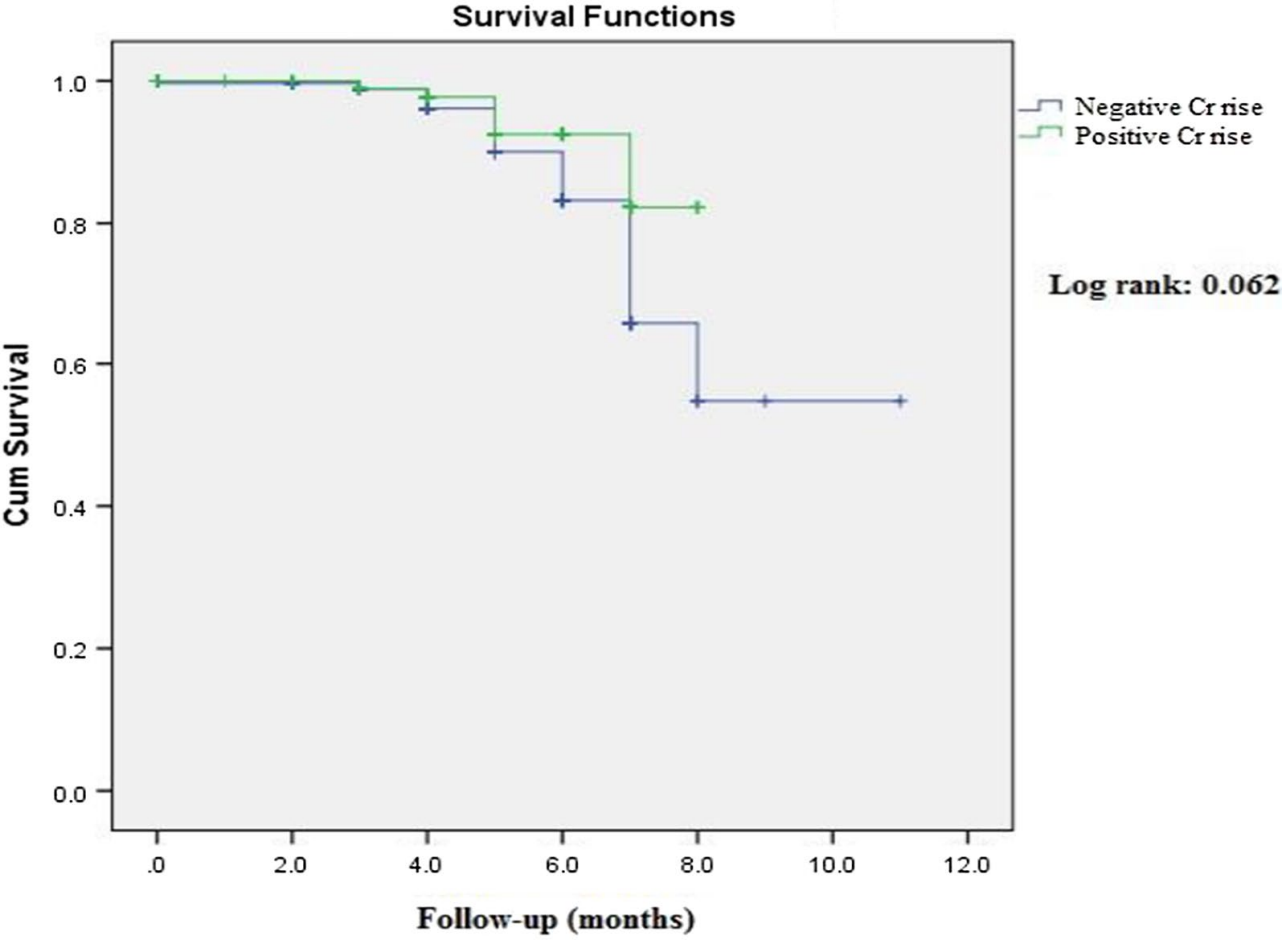
Kaplan–Meier survival curve of study population according to Cr rise status

Figure 2 depicts the Kaplan–Meier survival curve of the study population based on Cr rise status. Our findings revealed no significant association in mortality between patients with or without Cr rise during the hospitalization period (Log-rank: 0.062).

Univariate and multivariate-adjusted HR of CVDs death based on Cr rise is presented in Table 5. Although patients who experienced an increase in Cr levels had a reduction in mortality, this relation was insignificant in both univariate and multivariate-adjusted models (HR 0.50, 95% CI 0.23–1.08, P = 0.080 and HR 0.48, 95% CI 0.22–1.04, P = 0.065, respectively).

**Table 5:** Univariate and multivariable-adjusted Cox regression hazard ratio of cardiovascular disease mortality according to creatinine rise

Variable	Models	Creatinine rise		P
		Negative	Positive	
CVD mortality	Univariate	1.00	0.50 (0.23–1.08)	0.080
	Multivariate*	1.00	0.48 (0.22–1.04)	0.065

*CVD* cardiovascular diseases

*Adjusted for chronic obstructive pulmonary disease, hemoglobin, white blood cells, platelets, lymphocytes, neutrophils, and sodium

## Discussion

This study's main aim was to determine the relation between PLR with CVDs mortality, re-hospitalization rates, and serum Cr in ADHF patients referred with signs and symptoms of decompensation to the emergency department. We found that patients in the second PLR quartile (119 < PLR < 198) had a minimally reduced likelihood of mortality rather than those who had the lowest quartile. There was no significant association between this index and Cr in the study population. To the best of our knowledge, the current study is the first to investigate the relation between PLR and mortality among Iranian individuals suffering from ADHF. It has been reported that inflammation plays a major role in the pathogenesis of several diseases, including coronary artery disease and HF [24, 25]. Inflammatory status leads to activation of leukocytes resulting in the secretion of multiple cytokines, including interleukin 6 (IL-6), tumor necrosis factor α (TNF-α), and C-reactive protein (CRP). The cytokines mentioned above have corrosive effects on myocardial cells, causing decreased left ventricular pump function and subsequent HF occurrence [23, 26]. On the other hand, lymphocytes act as protective factors by expressing tissue inhibitors of metalloproteinase-1 [27]. HF-induced cortisol secretion due to activation of the hypothalamus–pituitary–adrenal (HPA) axis might cause decreasing lymphocyte counts, ultimately leading to declining survival rates among individuals with HF [21, 28]. This new biomarker is an inexpensive bedside blood index and could be done on all admitted patients. Moreover, because this indicator is a ratio, it is less variable than the platelets or lymphocyte counts alone and could be used more frequently in clinical settings. However, other studies are required for the determination of the exact usage of this possible prognostic factor.

Several studies were in agreement with our findings. For instance, *Durmus *et al*.* implemented a study to find the effect of PLR on mortality in decompensated HF. They recruited 56 patients with ADHF (age: 67.5 ± 12.6 years) and 40 age and sex-matched controls (age: 64.6 ± 8.5 years). The best cut-off value was reported to be 137.3. They followed their participants for 12.8 ± 7.6 months. They finally concluded that although PLR was significantly higher in HF patients than the controls, multivariable logistic regression failed to prove any great association in this regard (OR 0.993, 95% CI 0.976—1.010, P = 0.407) [21]. The mean age of the population in our study was quite the same. Furthermore, participants with different PLR categories had uniform distribution regarding chronic diseases, including diabetes mellitus or hypertension, in both studies. Additionally, *Pourafkari *et al*.* performed a retrospective study to investigate the potential roles of PLR on long-term mortality among those with acute HF. They gathered 554 admission data from 354 distinct patients. They classified participants according to PLR status into three tertiles, including tertile 1 (PLR < 137), tertile 2 (137 < PLR < 210), and tertile 3 (PLR > 210). After adjustment of all potential confounders, they figured out that neither tertile 2 nor tertile 3 had significant association with long-term survival (tertile 2/1: HR 1.243, 95% CI = 0.707–2.184, P = 0.449, tertile 3/1: HR 1.043, 95% CI = 0.497–2.188, P = 0.912) [29]. Another study on 551 patients with advanced HF who were classified into three groups according to PLR (< 150, 150–300, and > 300) indicated that PLR could not be used as an independent mortality predictor [30].

On the other hand, some other studies suggest different outcomes [19, 20, 31–33]. In *Ye *et al*.*’s study, 443 acute HF patients were enrolled and followed for 143.68 days to evaluate the prognostic value of PLR quartiles on clinical outcomes. After doing multivariate Cox proportional HR, they announced that third and fourth quartiles showed significant independent association with all-cause mortality (third quartile: HR 3.118, 95% CI = 1.668–5.386, P < 0.001, fourth quartile: HR 2.437, 95% CI = 1.302–3.653, P < 0.001) [31]. Patients in the highest PLR quartile (PLR > 177.17) were significantly older compared to the first quartile (73.54 ± 6.92 years vs. 71.25 ± 7.03 years, P = 0.045). Moreover, smoking status was distributed differently across quartiles of PLR, and these mentioned points should be taken into account for the generalization of their outcomes. Another study was implemented on 115 patients with a worrisome sign of HF, named acute cardiogenic pulmonary edema, with a mean follow-up duration of 20.8 ± 16.1 months. Patients were categorized into tertiles of the high, medium, and low PLR (> 194.97, 98.3 – 194.97, and < 98.3, respectively). After completion of follow-up duration, their findings suggested that higher tertiles were associated with higher total mortality (tertile 2/1: HR 2.730, 95% CI = 1.198–6.221, P = 0.017, tertile 3/1: HR 5.657, 95% CI = 2.467–12.969, P < 0.001). Although their study population had a quite similar mean age (69.3 ± 11.8 years) to ours, their small sample size and the recruitment of only a specific group of HF patients with probable worsen outcomes might limit the generalization of their findings [32]. *Turcato *et al*.* assessed the relation of PLR with 30-day mortality in 439 admitted patients with ADHF. The optimal cut-off point calculated by receiver operating characteristic curve analysis was reported to be 272.9, and patients were consequently classified into lower and higher groups. Their findings revealed that those patients with PLR of more than 272.9 had 5.89 (95% CI = 3.10–11.20, P < 0.001) and 3.22 (95% CI = 1.56–5.68, P < 0.001) times higher likelihood of 30-day mortality in comparison to those with lower PLR in the univariate and multivariate binary logistic regression models, respectively [33].

With regard to Cr rise, our findings were insignificant in terms of any association between PLR and renal function status. To the best of our knowledge, the current study is the first in the literature to assess this relation among HF patients. HF is now considered a systemic disease because it not only affects the heart itself but can also negatively affect other body organs, especially kidneys. On the other hand, renal dysfunction affects outcomes in HF sufferers and has been associated with increased cardiovascular mortality. Likewise, more than 40% of patients with HF concurrently suffer from chronic kidney disease [34, 35]. Therefore, the concept of cardio-renal syndrome seems to describe this interplay appropriately. This entity simply indicates that one organ dysfunction could lead to the impairment of another organ [35]. Several contributing factors have been suggested to be effective in this organ cross-talk, including inflammation resulted from circulatory inflammatory cytokines, hemodynamic factors, sympathetic autonomic nervous system, and renin–angiotensin–aldosterone system (RAAS) [35, 36]. These mentioned factors also have some interactions with each other so that activation of RAAS leads to increased sympathetic pathways resulting in inflammation. On the other hand, the inflammation itself might be one of the pivotal mechanisms in the activation of both the sympathetic nervous system and RAAS [35, 37, 38]. Due to previously proved cardiac and renal impairment in context of inflammation, PLR might negatively affect these organs, which might lead to increased mortality. This index might be practical in determining this kind of patient's prognosis with respect to renal function biomarkers [17, 39]. However, further studies are required in this regard.

This study was the first to assess this inflammatory biomarker's utility in Iranian individuals. However, several limitations are attributed to current research. A relatively small sample size and short follow-up duration might affect our reported findings. Moreover, this study was done in a single heart center, and caution should be made while interpreting the outcomes. We did not collect data on other inflammatory markers, including IL-6, TNF-α, and CRP. Other renal parameters, including glomerular filtration rate or fractional excretion of sodium, were not gathered to assess the possible effects of all renal indices on the relation between PLR and mortality. Finally, PLR data were not gathered at discharge time to assess any probable fluctuation during hospitalization.

In conclusion, despite growing literature suggesting that PLR has a prognostic value, our findings indicate that this blood index could not be independently used as a prognostic tool determining mortality as well as Cr rise among ADHF patients. However, one PLR quartile could partially predict CVDs mortality. Several future studies are necessary to define the exact worthiness of this bedside inflammatory index.

## Data Availability

The datasets generated during and/or analyzed during the current study are not publicly available due to confidential issues but are available from the corresponding author on reasonable request.

## Abbreviations

HF: Heart failure
PLR: Platelet to lymphocyte ratio
CVD: Cardiovascular disease
ADHF: Acute decompensated heart failure
PROVE/HF: Persian Registry Of cardioVascular diseasE/Heart Failure
IUMS: Isfahan University of Medical Sciences
COPD: Chronic obstructive pulmonary
BMI: Body mass index
ACEIs: Angiotensin-converting enzyme inhibitors
ARBs: Angiotensin receptor blockers
SBP: Systolic blood pressure
DBP: Diastolic blood pressure
LVEF: Left ventricular ejection fraction
BUN: Blood urea nitrogen
Cr: Creatinine
SD: Standard deviation
ANOVA: Analysis of variance
HR: Hazard ratio
OR: Odds ratio
SPSS: Statistical Package for Social Sciences
IL-6: Interleukin 6
TNF-α: Tumor necrosis factor α
CRP: C-reactive protein
HPA: Hypothalamus-pituitary-adrenal
RAAS: Renin angiotensin aldosterone system
